# A Handheld Metabolic Device (Lumen) to Measure Fuel Utilization in Healthy Young Adults: Device Validation Study

**DOI:** 10.2196/25371

**Published:** 2021-05-17

**Authors:** Kent Arnold Lorenz, Shlomo Yeshurun, Richard Aziz, Julissa Ortiz-Delatorre, James Robert Bagley, Merav Mor, Marialice Kern

**Affiliations:** 1 Department of Kinesiology San Francisco State University San Francisco, CA United States; 2 Metaflow Ltd Tel Aviv Israel

**Keywords:** resting metabolic rate, Lumen, ParvoMedics TrueOne 2400, validation, respiratory exchange ratio, metabolism, fuel utilization, indirect calorimetry, breath, lung, respiratory, young adult, measurement, testing

## Abstract

**Background:**

Metabolic carts measure the carbon dioxide (CO_2_) produced and oxygen consumed by an individual when breathing to assess metabolic fuel usage (carbohydrates versus fats). However, these systems are expensive, time-consuming, and only available in health care laboratory settings. A small handheld device capable of determining metabolic fuel usage via CO_2_ from exhaled air has been developed.

**Objective:**

The aim of this study is to evaluate the validity of a novel handheld device (Lumen) for measuring metabolic fuel utilization in healthy young adults.

**Methods:**

Metabolic fuel usage was assessed in healthy participants (n=33; mean age 23.1 years, SD 3.9 years) via respiratory exchange ratio (RER) values obtained from a metabolic cart as well as % CO_2_ from the Lumen device. Measurements were performed at rest in two conditions: fasting, and after consuming 150 grams of glucose, in order to determine changes in metabolic fuel usage. Reduced major axis regression and simple linear regression were performed to test for agreement between RER and Lumen % CO_2_.

**Results:**

Both RER and Lumen % CO_2_ significantly increased after glucose intake (*P*<.001 for both) compared with fasting conditions, by 0.089 and 0.28, respectively. Regression analyses revealed an agreement between the two measurements (*F_1,63_*=18.54; *P*<.001).

**Conclusions:**

This study shows the validity of Lumen for detecting changes in metabolic fuel utilization in a comparable manner with a laboratory standard metabolic cart, providing the ability for real-time metabolic information for users under any circumstances.

## Introduction

Indirect calorimetry (metabolic cart), which is currently the preferred method for determining metabolic fuel utilization, measures the carbon dioxide produced (VCO_2_) and oxygen consumed (VO_2_) when breathing. The ratio between VCO_2_ and VO_2_ is the respiratory exchange ratio (RER), which provides insight into the relative contribution of carbohydrates and lipids to overall energy expenditure [1,2]. Though indirect calorimetry is not invasive, this method is time-consuming (up to 40 minutes), only available in test laboratory settings, and requires technical and physiological expertise for handling the metabolic cart and interpretation of the metabolic data obtained.

**Figure 1:**
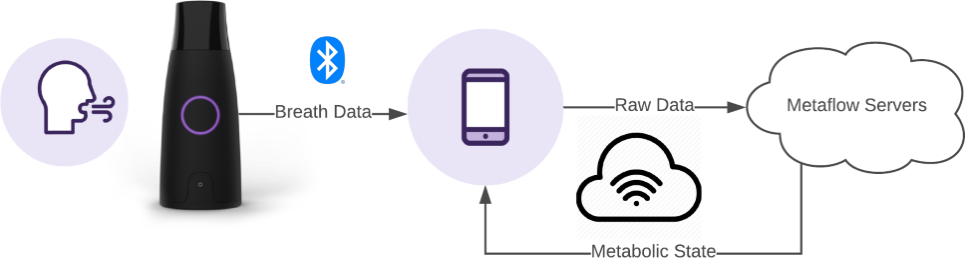
A schematic representation of the Lumen device and app.

Metaflow Ltd developed Lumen, a novel metabolic fuel utilization breathalyzer, which is a personalized handheld device that provides an individual’s metabolic state in real time by measuring CO_2_ from exhaled breath (Figure 1). The device indirectly measures metabolic fuel usage via a CO_2_ sensor and a flow sensor to determine the rate of CO_2_ production from a single breath maneuver. The % CO_2_ in the exhaled volume of air is determined from a specific breathing maneuver with a breath hold of 10 seconds. This concept is based on the fact that oxygen consumption is stable under resting conditions [3]; thus, a change in metabolic fuel use will generally be represented by changes in CO_2_ production. For carbohydrate oxidation, more carbon dioxide is produced relative to the consumption of oxygen. For fat oxidation, less carbon dioxide is produced [4]. The use of a smartphone app enables the user to track metabolic status outside of physiologic test laboratories.

Previous exploratory studies for algorithmic development of Lumen were performed to compare the Lumen measurement to the metabolic cart. In this study, we aim to evaluate agreement between the Lumen measurement and that of the metabolic cart in healthy participants before and after glucose ingestion under stable resting conditions.

## Methods

### Participants

A total of 54 healthy volunteers reported to the Exercise Physiology Laboratory in the Department of Kinesiology at San Francisco State University to participate in this study. Inclusion criteria were being aged between 18-45 years with a BMI less than 30 kg/m^2^. Exclusion criteria were participation in high-intensity aerobic training or having a known cardiovascular, pulmonary, and/or metabolic disease. The study was approved by the university’s Institutional Review Board for Human Subjects, and written informed consent was obtained from each participant before testing.

### Study Design

Participants were recruited and their height and weight were measured using a stadiometer and Seca scale (Seca). If they met the BMI criteria, they were provided their own Lumen device, which was labeled with their unique identification number. The Lumen device was paired and synchronized to the participant’s smartphone together with the Lumen app. Participants practiced the Lumen breathing technique while supervised and took the device home for a further familiarization period in order to show proficiency with the device and app. They were instructed to perform Lumen metabolic measurements for at least 30 sessions, with each session consisting of 3 breath maneuvers, and to complete 3 sessions at different time points each day. After the minimum amount of home breath sessions were collected, participants were scheduled for the study laboratory measurement day. All participants came to the test laboratory between 7 AM and 11 AM after a 12-hour fast and had abstained from any form of physical activity (other than walking).

On the laboratory testing day, blood glucose samples were taken by sterile finger prick blood sample and measured by a glucometer (OneTouch, LifeScan Inc). For the indirect calorimetry measurement, the participant had to lay down in supine position on a padded examination table, where a rigid clear plastic canopy with a comfortable, flexible seal was placed over the head and upper part of the torso. Once the metabolic cart measurement was completed, the participant was seated in a comfortable chair. After 5 minutes of rest, they were asked to perform two Lumen breath sessions (5-minute break between each session). The first Lumen session immediately after the metabolic cart measurement was used for data analysis. In case of an invalid first session (difference between breaths >0.2% CO_2_), the second session was used for analysis.

Once finished, participants were asked to drink 150 grams of a glucose solution (3 servings of 50 grams with 20-minute intervals between each serving). Subsequently, 45 minutes after the intake of the first drink (corresponding to 5 minutes after finishing the last serving), their glucose levels were reassessed, and the same assessment procedures as during the fasted state before the glucose intake were repeated. Participants were removed from the analysis if they were unable to finish all glucose drinks.

### Metabolic Cart

RER was analyzed using a calibrated TrueOne 2400 metabolic cart (ParvoMedics), which was previously determined to provide a valid measurement for RER with 5% coefficient of variation [5]. This system uses a paramagnetic oxygen analyzer and infrared carbon dioxide analyzer with a Hans Rudolph heated pneumotach. The ParvoMedics system was warmed up for at least 60 minutes each day before testing to ensure accurate and stable readings. The gas analyzers and flow sensor were calibrated as per manufacturer’s recommendations: calibration of the analyzers was performed using a high-precision gas mixture (O_2_, CO_2_, remainder N_2_) and calibrated and accepted with a <0.1% error with the calibration gas. Flow and volume were calibrated using a calibrated 3 L syringe (Hans Rudolph, model 5530) to ≤1% error. In addition, verification of the calibration process was performed to ensure stability of the system. The ambient temperature was kept between 22 °C and 26 °C in the test laboratory. Relative humidity was maintained stable at roughly 60%. Once calibration was acceptable and complete, a ventilated hood with subject cover was placed over the participant’s head and positioned around the upper torso area to ensure no air could escape from the hood. The participants were required to stay awake during the measurement procedure. The hood ventilation was measured during the recording, and CO_2_ and O_2_ concentrations were measured from it. VCO_2_ and VO_2_ parameters were calculated and taken as 30-second averages. For this study, we defined the subject steady-state metabolic measurement based on observed variations in the VO_2_ and VCO_2_ of less than ≤5% coefficient of variation for a period of at least five consecutive minutes, with a subsequent RER stability of 2.5% in a fasted state and 3.7% after glucose consumption, in a similar manner to previous studies [6]. Inability to meet these criteria resulted in removal of the data from the analysis.

### Lumen

Lumen is a device designed to be calibration-free, with a warm-up time of less than 10 seconds and the CO_2_ sensor taking into account the room CO_2_ concentration during every measurement. During the measurement day, participants completed 2 sessions of 3 Lumen breaths each after the metabolic cart measurement. The Lumen breathing maneuver consists of three phases, starting from the end of a normal expiration (functional residual capacity). The participant takes a deep breath in through the Lumen device, followed by a 10-second breath hold. Afterward, the subject exhales through the Lumen device, with a steady exhalation flow to at least the starting level of the maneuver. In order to confirm repeatability, breaths are taken in triplicate for each session. The Lumen smartphone app guides the participant through each phase of the Lumen maneuver. Each Lumen session was repeated after a 5-minute pause interval. Validity of breath maneuvers was systematically evaluated by the Lumen app. Inability to perform valid Lumen breath measures resulted in removal of the data from the analysis.

### Statistical Analyses

All variables were tested and visualized for normal distribution before the tests.

To evaluate the changes after glucose intake, two-tailed paired parametric *t* tests were performed for blood glucose levels, RER levels, and Lumen % CO_2_ before and after glucose intake.

For agreement validation, major axis regression (Deming method) was performed to compare RER of the metabolic cart and % CO_2_ from the Lumen device [7]. As RER and % CO_2_ are in different units, the analysis is identical to ordinary least products regression (also known as reduced major axis regression), which is the most suitable analysis for comparison between two methods of measurement [8]. Moreover, a simple linear regression (ordinary least squares) was performed to determine the ability to predict Lumen values from the gold-standard value of RER.

Statistical analyses were performed using GraphPad Prism 8 (GraphPad Software Inc). The threshold for significance was set at *P*<.05.

### Ethics Statement

This study was approved by San Francisco State University’s Institutional Review Board for Human Subjects, and written informed consent was obtained from each participant before testing.

## Results

**Figure 2:**
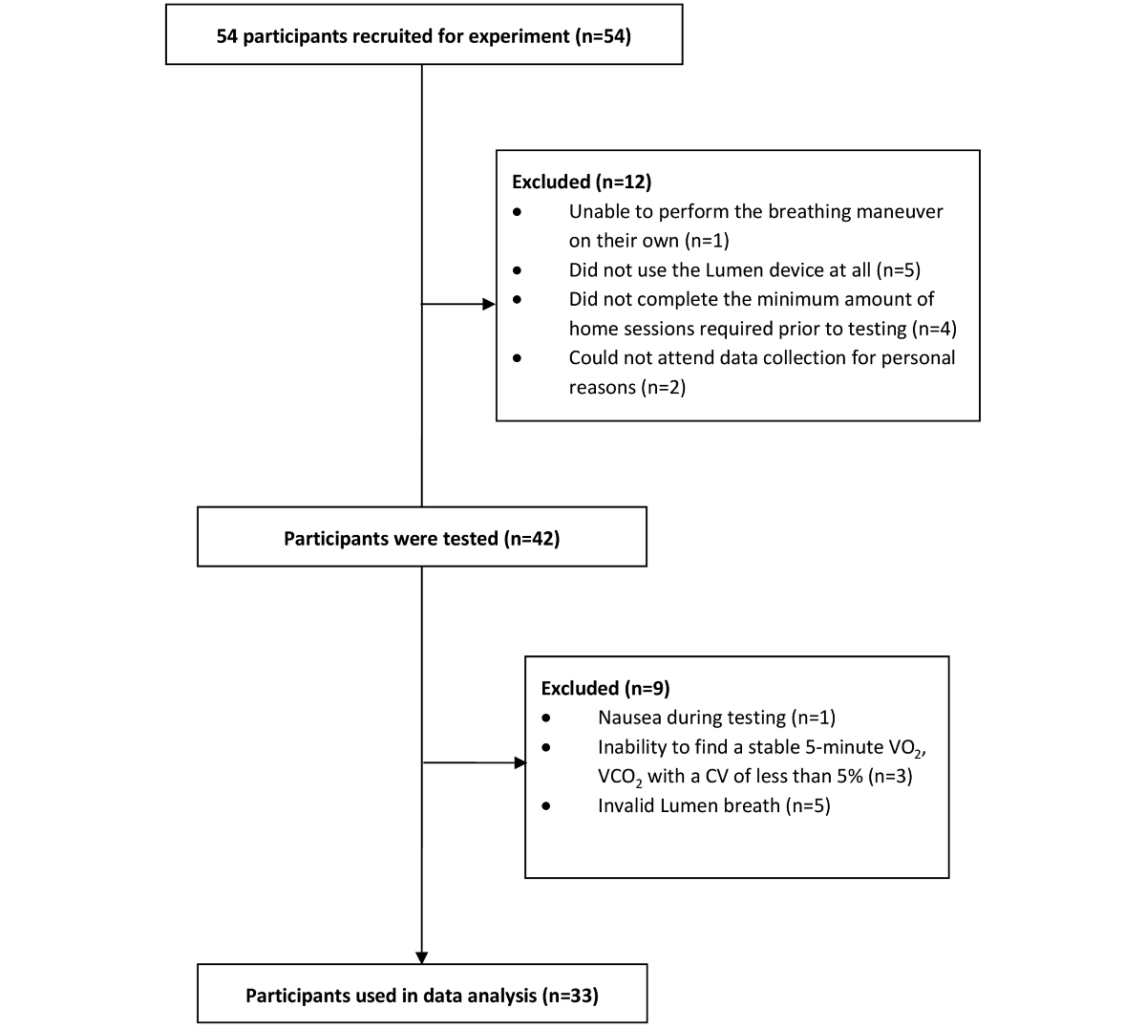
Consolidated Standards of Reporting Trials (CONSORT) flow diagram. CV: coefficient of variation.

From the original 54 participants recruited, 12 were excluded prior to laboratory testing and 9 had to be excluded during the testing day for failing to meet the inclusion criteria as detailed in the methods section: 1 participant was unable to consume all glucose drinks due to nausea, 3 participants did not achieve 5 minutes of stable metabolic cart measurement (coefficient of variation <5% in VO_2_ and VCO_2_), and 5 participants were unable to perform a valid Lumen measurement (Figure 2). Characteristics of the final 33 participants are presented in Table 1.

**Table 1:** Descriptive statistics of study participants.

Gender	Count	Age (years), mean (SD)	Weight (kg), mean (SD)	Height (cm), mean (SD)	BMI (kg/m^2^), mean (SD)
Male	17	24.0 (3.0)	73.7 (10.2)	171.7 (7.8)	24.9 (2.5)
Female	16	22.3 (4.5)	59.1 (6.4)	160.9 (5.5)	22.9 (2.6)
Total	33	23.1 (3.9)	66.2 (11.1)	166.1 (8.6)	23.9 (2.7)

**Figure 3:**
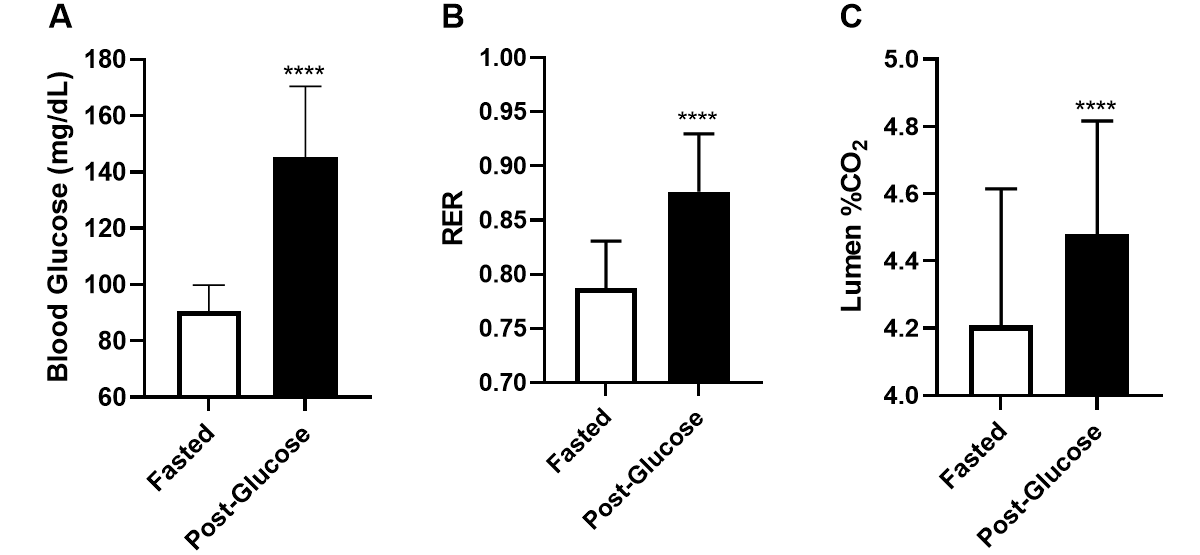
Changes in blood glucose as determined by (A) blood glucose test, (B) RER, and (C) Lumen % CO_2_. Data are presented as mean (SD). N=33 for each state. **** indicates *P*<.001. RER: respiratory exchange ratio.

Blood glucose levels increased from 90.6 (SD 9.2) mg/dL to 145.2 (SD 25.3) mg/dL as a result of glucose intake (*t_32_*=11.04, *P*<.001; Figure 3A). RER levels increased from 0.787 (SD 0.043) to 0.876 (SD 0.053) in response to glucose intake (*t_32_*=10.84, *P*<.001; Figure 3B). Moreover, Lumen CO_2_ concentrations significantly rose from 4.20 (SD 0.4) to 4.48 (SD 0.34; *t_32_*=5.978, *P*<.001; Figure 3C). These analyses have confirmed the ability of both the metabolic cart and Lumen to detect changes in metabolic fuel utilization.

**Figure 4:**
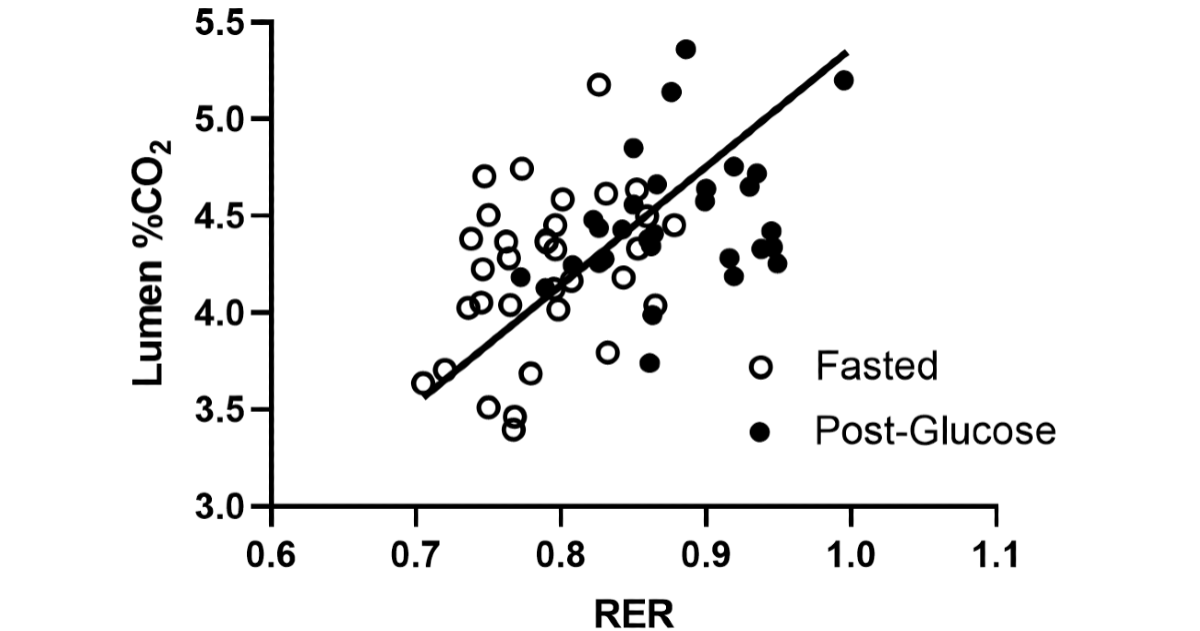
Reduced major axis regression of RER from the metabolic cart and Lumen % CO_2_ measurements for metabolic activity. N=33 for each state. RER: respiratory exchange ratio.

To test for agreement between RER units from the metabolic cart and % CO_2_ from Lumen, reduced major axis regression was performed [9]. It revealed a significant relationship between RER and Lumen % CO_2_ (*F_1,63_*=18.54, *P*<.001, y=6.111x–0.7445, x-intercept=0.1218; Figure 4). This analysis confirmed the agreement between Lumen % CO_2_ and metabolic cart RER, with a systemic bias as a result of the nature of the different units.

**Figure 5:**
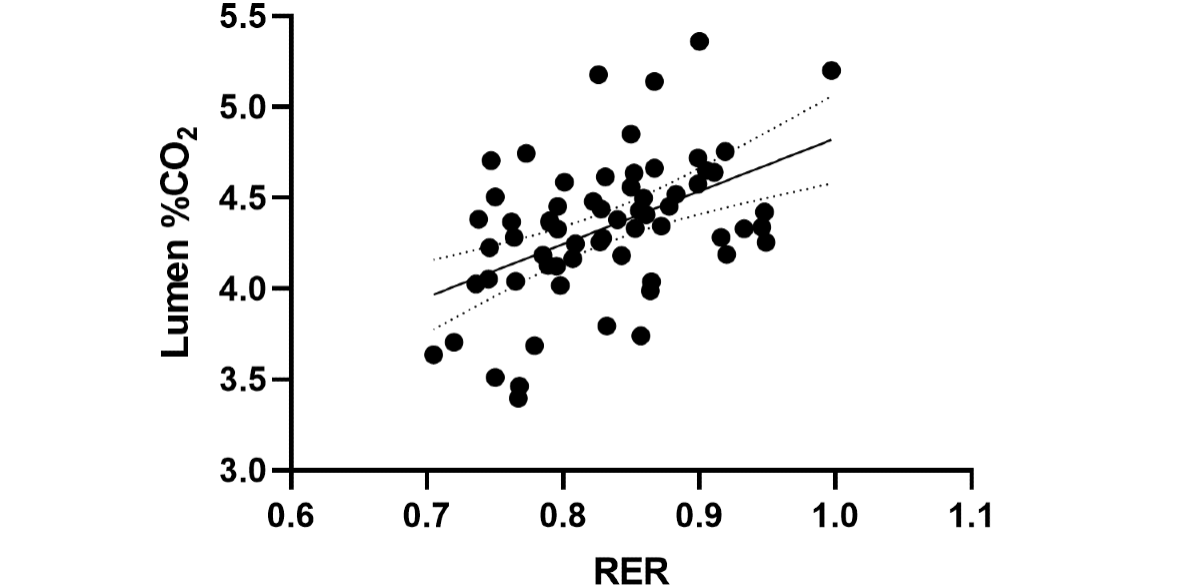
Ordinary least squares regression of RER and Lumen % CO_2_. N=33 for each state. RER: respiratory exchange ratio.

To determine the ability of metabolic cart RER to predict Lumen % CO_2,_ ordinary least squares regression was performed to estimate Lumen values from RER measures, with the assumption that RER is an accurate independent measure, to predict Lumen % CO_2_. A significant model effect was present (*F_1,63_*=18.54, *P*<.001, *R*^2^=0.2274; Figure 5). The RER parameter estimate indicated that for every 1-unit increase in RER, a 2.914-unit increase (SE 0.6767) in Lumen % CO_2_ is expected. Since a full unit increase in RER is not a plausible outcome, this parameter estimate can be interpreted similarly by a 0.1-unit increase in RER (eg, 0.7 to 0.8) to produce a 0.2914-unit increase in Lumen % CO_2_.

## Discussion

### Principal Findings

This study evaluated the ability of the Lumen device to assess changes in the body’s metabolic fuel utilization in healthy young adults compared to the indirect calorimetry metabolic cart measurement. Our results show that Lumen CO_2_ levels are in agreement with RER values from the metabolic cart, which correspond to relative changes in metabolic fuel utilization.

Both Lumen CO_2_ levels and metabolic cart RER showed significant increases in metabolic levels as a result of glucose intake in healthy individuals in resting conditions (Figure 3). These results can be expected, as cells using more carbohydrates as fuel produce more CO_2_ relative to O_2_ consumption compared to cells metabolizing fat. The ratio between CO_2_ production and O_2_ consumption in this process is known as the respiratory quotient (RQ) or RER. RQ and RER vary depending on the energy source of the cell (carbohydrate versus fat), and the acronyms are commonly used interchangeably [2,10,11]. In resting conditions, oxygen consumption is fairly stable [12,13], meaning that participants’ changes in RQ are due to changes in CO_2_ production. This is the underlying concept of the Lumen device, enabling it to track changes in metabolic fuel utilization. For that reason, it was important to ensure that participants in this study were at rest before and during their measurements.

Reduced major axis regression revealed an agreement between RER and Lumen CO_2_ levels (Figure 4). This analysis enables us to test for agreement between methods with different units and verify the validity of the Lumen device with a metabolic cart. It demonstrates the ability of the Lumen device to provide equivalent results to the metabolic cart in assessing metabolic fuel utilization.

Furthermore, the results from the simple linear regression predicting Lumen % CO_2_ using RER values suggest that, while there is measurement agreement between the Lumen % CO_2_ and RER, the proportion of variance remains low (Figure 5). Thus, Lumen can be seen to be an effective instrument for monitoring individual changes in metabolic responses (within-subject consistency), rather than a substitute for the metabolic cart (between-subject precision).

Evidence suggests that the assessment of RER can be beneficial for multiple applications, such as nutrition, diabetes prevention, or weight management [14]. It has previously been shown that RER could be a prognostic marker of weight loss and a predictor of weight gain [15,16]. Moreover, minute-to-minute RER measured in a respiratory chamber calorimeter showed that the slopes of RER were different in response to different dietary interventions [17]. However, although RER is currently the preferred method for determining metabolic fuel, it is a time-consuming, uncomfortable, and costly and impractical tool for real-time day-to-day assessments of metabolic activity. In contrast, the Lumen device is small, mobile, user-specific, and relatively cheap, and delivers the outcome immediately to the user and enables real-time decisions.

### Limitations

This study is the first to show agreement between Lumen % CO_2_ and RER. However, it is important to note that participants in this study were young (mean age 22.4 years) and healthy individuals. With increasing age, metabolism changes, as can be seen in various metabolic cart studies [18-20]. Future studies will need to examine whether RER metabolic cart levels correspond to Lumen CO_2_ levels in older subjects and those with metabolic conditions.

Unlike the metabolic cart, the Lumen device does not measure oxygen consumption. Accordingly, the Lumen measurement should be performed under resting conditions with stable VO_2_, allowing the correct interpretation of changes of % CO_2_ as changes in metabolic state.

In addition, results from this study showed a high peak of blood glucose levels 45 minutes after glucose intake (5 minutes after the third drink), whereas both RER and Lumen % CO_2_ showed a more moderate increase in levels. It is possible that the metabolic cart and Lumen measurements were performed too early, as it may be that in some of our participants, the peak glucose levels occurred more than 45 minutes after ingestion; thus, it was not yet fully metabolized [21].

### Conclusions

In summary, Lumen can provide valid information regarding an individual's metabolic state, and in agreement with results from the metabolic cart. Unlike the metabolic cart, Lumen measurement can be performed anywhere, anytime, without the need for a specialized laboratory, equipment, and technical staff. The Lumen device is able to detect changes in metabolism due to dietary intake, similarly to the metabolic cart. The capability of taking metabolic measurements continuously outside of laboratory settings can provide new insights about the metabolic state of an individual so as to obtain further knowledge and understanding about metabolism and nutrition.

## Abbreviations

RER: respiratory exchange ratio
RQ: respiratory quotient
VCO_2_: carbon dioxide production
VO_2_: oxygen consumption
